# Prospective two-year subsidence analysis of 100 cemented polished straight stems - a short-term clinical and radiological observation

**DOI:** 10.1186/s12891-016-1247-9

**Published:** 2016-09-17

**Authors:** Wolf Siepen, Lukas Zwicky, Karl Kilian Stoffel, Thomas Ilchmann, Martin Clauss

**Affiliations:** 1 Department for Orthopaedics and Trauma Surgery, Kantonsspital Baselland, CH-4410 Liestal, Switzerland; 2 LEONARDO, Hirslanden Klinik Birshof, Münchenstein, Switzerland

**Keywords:** Cemented, EBRA-FCA, RM pressfit, Straight stem, Subsidence, twinSys

## Abstract

**Background:**

Cemented stems show good long-term results and the survival of new implants can be predicted by their early subsidence. With EBRA-FCA (Femoral Component Analysis using Einzel-Bild-Röntgen-Analyse) early subsidence as an early indicator for later aseptic loosening can be analysed. For the cemented TwinSys stem mid- and long-term data is only avalible from the New Zeeland Arthroplasty register, thus close monitoring of this implant system is still mandatory.

**Methods:**

We conducted a 2 year follow up of 100 consecutive hybrid THA (Total hip arthroplasty) of a series of 285 primary THA operated between Jan 2009 und Oct 2010. These 100 received a polished, cemented collarless straight stem (twinSys®, Mathys AG® Bettlach, Switzerland) with an uncemented monobloc pressfit cup (RM pressfit®, Mathys AG® Bettlach, Switzerland). The other patients were treated with the uncemented version of this stem and the same cup. Clinical (Harris Hip Score) and radiological (ap and axial x-rays, cementing quality according to Barrack, alignment) outcomes besides an EBRA-FCA subsidence analysis were performed.

**Results:**

Median age at operation was 78 (68 to 93) years. 5 patients died in the course of follow-up unrelated to surgery. The KM (Kaplan-Meier) survival at 2 years for the endpoint reoperation for any reason was 94.9 (95 % confidence interval 90.6–100 %). Survival for the endpoint aseptic loosening at 2 years was 100 %.

The HHS (Harris Hip Score) improved from 56 (14–86) preoperatively to 95 (60–100) 2 years after the operation. Cementing results were judged 47 % Grade A, 45 % Grade B and 7 % Grade C.

Osteolysis was found in 2 stems without clinical symptoms or correlation to subsidence or cementing quality. The EBRA-FCA analysis showed an average subsidence of -0.30 mm (95 % CI -0.5 mm to -0.1 mm). 11 patients showed a subsidence of more than 1 mm. In this group one patient showed a subsidence of 1.5 mm and one of 3.1 mm without further radiological changes.

**Conclusions:**

The twinSys stem showed excellent clinical and radiologic short term results at 2 years follow-up and seems to be a reliable implant.

## Background

Long-term success of a cemented stem depends on the longevity of the cement-bone and the cement-prosthesis interface. Early subsidence above a certain threshold is highly predictive for failure of cemented stems. Different cut-off values, depending on the means of measurement, are described [1–4]. These different measurements can be performed with plain radiographs, EBRA-FCA (Femoral Component Analysis using Einzel-Bild-Röntgen-Analyse) or RSA (Roentgen Stereophotogrammetric Analysis). Plain radiographs have the lowest accuracy and RSA offers the highest accuracy with EBRA-FCA being in the middle [5].

Two different design concepts, namely composite-beam” (“shape-closed”) and “load-tapered” (“force-closed”), are described for the fixation of a cemented stem [6]. A classic composite-beam stem is the Muller straight stem, excellent long-term data has been shown [7]. For load-tapered stems, the Exeter stem is the typical example. The double tapered design allows lodging as a wedge in the cement when axially loaded reducing peak forces [1]. Some initial subsidence is observed until radial compressive forces are transferred to the cement and conducted as hoop stress to the bone equal the axial loading in an equilibrium and therefore terminating futher subsidence hence contributing to the final position of a stable implant [1]. The Exeter stem is one of the most successful stem designs with outstanding long-term results [8–13].

The cemented twinSys stem was designed according to the load-taper design philosophy but compared to the Exeter it is a triple taper design. So far no track record exists for this implant system, despite promising results form the New Zealand Joint Registry Annual Report 2015 [14] with the cemented twinSys used as one of the top 10 implants and an overall revision rate of 0.58 per 100 component years in combination with the RM cup as used in our collective.

The twinSys stems also exists as an uncemented version, allowing the surgeon the use of both stems with the same instrumentation and granting freedom to intraoperatively change the fixation method besides reducing the stock material in the theatre. Therefore close monitoring of this new implant is mandatory until long-term data with high case numbers exists which can confirm the longevity with clinical long-term results.

Aim of this study was to analyse the short-term survival, early subsidence and radiological changes of the cemented twinSys straight stem.

## Methods

Between Jan 2009 and Oct 2010 a total of 285 primary total hip arthroplasty (THA) were performed at our institution, 100 (97 patients) of them were operated with the cemented twinSys straight stem and followed prospectively after 6, 12, 52 and 104 weeks. Median age at operation was 78 (68 to 93) years. 51 stems were implanted in female patients, 53 on the right side. Diagnoses were 79 osteoarthritis, 6 osteonecrosis and 15 femoral neck fractures. The other 185 primary THA during the study period were operated with the uncemented twinSys stem. Operations were performed or supervised by two senior consultants (TI, MC). Data analysis and EBRA-FCA was performed by an independent surgeon (WS) not beeing involved in the operations nor follow-ups. All patients agreed to participate in the study and approval of the local ethics committee (EKNZ (Ethikommission Nordwest Schweiz) 2015-125) was obtained. No patient was lost to follow-up.

The cemented twinSys stem has a polished surface (mean roughness Ra 0.4 μm) and a triple taper with an angle in the lateral projection of 4° proximal und 1.5° distal and 5° in the ap projection. A standard and a lateralised version are available, whith the lateralisied version used in 45 of our cases as needed to reconstruct the offset and joint geometry. 97 stems were combined with a cementless RM pressfit cup (Mathys AG Bettlach, Switzerland), 3 stems with a Muller acetabular reinforcement ring (ARR) and a cemented PE cup. The RM pressfit® cup is made out of standard UHMW-PE, with a 28 or 32 mm articulation. The new RM vitamys® cup, made out of highly crosslinked PE, offers a 36 mm articulation, which we use nowadays if patients are at higher risk for dislocation. For all hips a ceramic head (Bionit 2®, Mathys AG Bettlach, Switzerland) was used, 35 heads had a 28 mm and 65 a 32 mm diameter.

All patients were operated in the routine setup of a university affiliated teaching hospital. Implant size, position and leg length were planned with a digital planning tool (AGFA® Orthopaedic Tools, Agfa HealthCare. N.V, Mortsel, Belgium) prior to surgery. 22 patients were operated in a supine position through a direct lateral transgluteal approach, and 78 with an anterior MIS approach as recently published [15]. Stems were cemented with a third-generation cementing technique with (Synplug®, Mathys AG Bettlach, Switzerland), cement syringe, vacuum-mixing, jet lavage but no proximal sealing using Palacos® G bone cement (Hereaus Medical, Dübendorf, Switzerland). Patients were mobilised either on the day of surgery or the day after with full weight bearing. Crutches were advised for comfort as needed for 6 weeks.

### Clinical evaluation

Clinical follow-up included a standardised examination, using the Harris Hip Score (HHS) [16] at all time points.

### Radiological evaluation

**Fig. 1:**
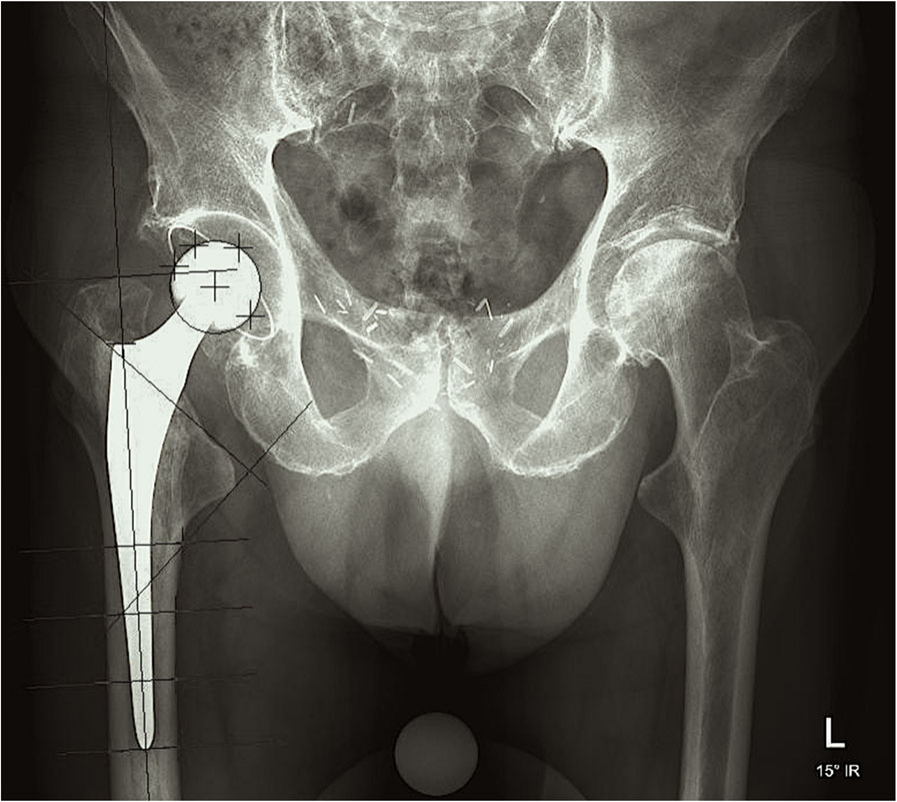
Distribution of the measuring points used to measure and calculate the subsidence in the EBRA-FCA software

Standardized digitalized radiographs of the pelvis (patient in supine position, centered on the symphysis, focus film distance 120 cm) were taken at 1 week, 12 weeks, 1 and 2 years postoperatively. The quality of the cement mantle was rated according to Barrack [17]. Varus/valgus alignment of the stem was measured on the postoperative ap radiograph, a deviation of more than 3° was defined as malalignment [18]. Debonding was defined as a radiolucent line at the prosthesis-cement-interface not visible on the first postoperative radiograph [18]. Osteolysis was defined as a progressive, newly developed endosteal bone loss with a diameter greater than 3 mm with an either scalloping or bead-shaped lucency at the cement-bone-interface [3]. Debonding and osteolysis were manually measured on the plain radiographs and reported accoording to their location in the Gruen zones [19]. Subsidence of the stem was measured using the software based EBRA-FCA method. For this method a minimum of 4 comparable digitalized x-rays are needed. An electronic coordinate system is placed on the x-rays to localize cup, stem and certain landmarks (Fig. 1). Compared to e.g. RSA no further markers have to be implanted and routine follow-up radiographs can be used. Therefore it’s an ideal tool to detect early subsidence or migration in huge cohorts [2, 3, 20].

Cup inclination was manually measured on the plain radiographs using the interteardrop line. Osteolysis around the cup was rated according to the zones described by DeLee and Charnley.

### Statistics

A Shapiro-wilk test was used to test for normal distribution of the data. As data were non normal distributed, median and range was used to describe the data.

For comparison of the data we used either a Mann-Whitney- or Chi-square test. Paired data were tested using a Wilcoxon signed rank test. Implant survival was calculated using Kaplan-Meier survival analysis for the endpoints aseptic loosening of the stem and reoperation for any reason. A *p*-value < 0.05 was considered significant. IBM SPSS Statistics 23 was used for statistical analysis.

## Results

### Survival analysis

**Fig. 2:**
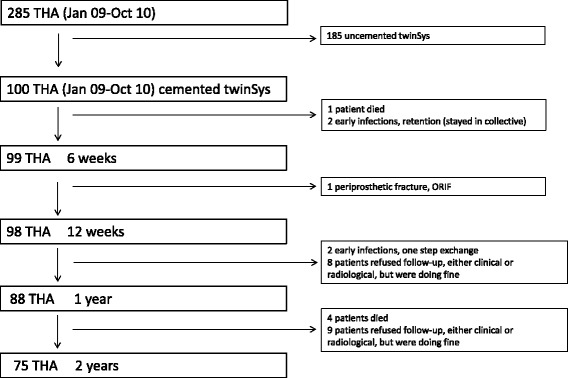
CONSORT flow chart of the included patients and the follow-ups

No patient was lost to follow-up. 17 patients had an incomplete follow-up missing either a clinical or x-ray follow-up, none had been revised. 5 patients died during the first 2 years unrelated to surgery (median 379 days, range 7–684 days). 2 hips had an early infection (<4 weeks postoperative) and were treated with debridement and implant retention (DAIR), both were free of infection at final follow-up. Another 2 patients had a late chronic infection (CNS 4 months, and *P. acnes* 8 months) and were successfully treated with a one-stage exchange. 1 patient sustained a periprosthetic fracture (Vancouver type B1) 3 months after surgery due to a fall, and was treated with osteosynthesis. 1 female patient had two early dislocations (4 and 5 weeks postoperative, 32 mm head, anterior approach) during night rest and was treated with closed reduction and a brace for 6 weeks. Afterwards she had a uneventful follow-up without further (sub-)luxations. Radiographs showed a normal alignment of the implants, thus the reason for dislocation remains unclear. Exact patient flow is shown in the CONSORT flow chart (Fig. 2).

The KM survival at 2 years for the endpoint reoperation for any reason was 94.9 (95 % confidence interval 90.6–100 %). Survival for the endpoint aseptic loosening at 2 years was 100 %.

### Clinical outcome

The HHS (Harris Hip Score) improved from 56 (14–86) preoperatively to 95 (60–100) 2 years postoperatively (Table 1). There was no difference in HHS scores between the two approaches (*p* = 0.91).

**Table 1:** Harris Hip Score during the time line, differentiation between transglutral an anterior (MIS) approach

Time point	HHS all	Transgluteal	MIS	*p*-value
preOP	56 (14–86)	57 (20–86)	56 (14–85)	0.64
6 weeks	82 (49–100)	75.5 (49–94)	84 (51–100)	0.06
12 weeks	93 (66–100)	87.5 (70–100)	94 (66–100)	0.8
1 year	99 (25–100)	99 (75–100)	99 (25–100)	0.59
2 year	95 (60–100)	95 (79–100)	95 (60–100)	0.91

### Radiological outcome

Seventy-three stems had a complete radiological follow-up consisting of 4 radiographs and were free of complications. 17 patients had an incomplete radiological follow-up. They were doing fine, but refused follow-up examinations or radiographs. 5 patients were revised for the reasons mentioned above and 5 patients deceased. One of these died before having post-op radiographs due to a gastro-intestinal bleeding.

Cementing quality (*n* = 99) was rated grade A in 47 stems, grade B in 45, grade C in 7 and grade D in 0 stems. There was no influence on cementing quality if the operation was performed by a consultant or a resident in training (*p* = 0.82).

Alignment (*n* = 99) was neutral for 70 stems, 15 in varus and 14 in valgus, which did not change during follow-up.

Osteolysis around the stem was seldom and seen in 2 stems in Gruen zone 7 and was independent from cementing quality or stem alignment. No debonding was noticed.

### EBRA-FCA analysis

Fifty-seven of 73 hips (78 %) with a complete radiological follow-up could be analysed with EBRA-FCA. The average subsidence was -0.3 mm (95 % CI -0.5 mm to -0.1 mm). 11 stems showed subsicence above 1 mm (Table 2). The stem with a subsidence of 1.5 mm had a cement mantle Barack grade A, neutral stem alignment and showed no further radiological changes (osteolysis or debonding) at final follow-up. The other patient with a subsidence of 3.1 mm had a cement mantle Barrack grade B, neutral stem alignment and also showed no other radiological abnormalities, but was succesfully treated with DAIR due to an early infection 4 weeks after implantation. There was no correlation between cementing quality and subsidence (*p* = 0.179) or osteolysis and subsidence (*p* = 0.634).

**Table 2:** Distribution of the subsidences measured by EBRA-FCA and the corresponding details about alignemnt, cementing quality and offset

CRI	Patient	Subsidence EBRA-FCA (mm)	Alignement	Cementing Quality (Barrack)	Standard/Lateralised Offset
195	1	1.01	Valgus	A	Lateralised
326	2	1.04	Neutral	A	Lateralised
182	3	1.23	Neutral	C	Lateralised
315	4	1.26	Neutral	A	Lateralised
347	5	1.31	Neutral	B	Lateralised
403	6	1.32	Neutral	A	Standard
338	7	1.35	Valgus	A	Standard
324	8	1.37	Neutral	A	Lateralised
345	9	1.44	Neutral	A	Standard
244	10	1.51	Neutral	A	Standard
288	11	3.08	Neutral	B	Standard

### Cup

The median cup inclination was 38° (range 11°–55°, 10–20°: *n* = 1, 21–30°: *n* = 9, 31–40°: *n* = 46, 41–50°, *n* = 38, 51–60°: *n* = 5). No progessive osteolysis were noted around the cup. No cup was revised for aseptic loosening or malpositioning.

## Discussion

We present a prospective study with a clinical and radiological analysis of 100 consecutive cemented twinSys stems including an EBRA-FCA analysis of 57 stems. Survival for aseptic loosening after 2 years was 100 % and 94.9 (95 % confidence interval 90.6–100 %) for all reasons of revision. This is comparable to survival rates of other well known and successful cemented systems in larger multi-surgeon series [7, 11, 21] as shown in Table 3. A strength of the study is the complete follow-up of all patients even though some patients missed or declined to come for single follow-up appointments because of a lack of clinical complaints.

**Table 3:** Overview of the different implants and their long-term survival for aseptic lossening

Author	Year	Type	Implant	Fixation type	Survivalrate and time
Clauss M [7]	2009	Follow-up	Müller straight stem	Shape-closed	87 % survival 20 years for aseptic loosening
Espehaug B [9]	2009	Register	Exeter	Force-closed	90 % survival 20 years for aseptic loosening
			Titan	Shape-closed	93 % survival 19 years for aseptic loosening
			Spectron	Shape-closed	90 % survival 17 years for aseptic loosening
Ling RS [10]	2009	Follow-up	Exeter	Force-closed	93,5 % survival 33 years for aseptic loosening
Makela K [11]	2008	Register	Exeter	Force-closed	>90 % survival 15 years for aseptic loosening
			Müller straight stem	Shape-closed	>90 % survival 15 years for aseptic loosening
Ogino D [21]	2008	Register	Lubinus	Shape-closed	95,5 % survival 23 years for aseptic loosening
			Exeter	Force-closed	95 % survival 15 years for aseptic loosening

### Clinical outcome

Our clinical results are comparable to normal short-term results of other successful implants. The increase of the functional status combined with a high rate of subjective satisfaction of patients receiving THA is a normal development for the postoperative course and a main reason for the high success rate and patient satisfaction for this operative procedure being the operation of the 20^th^ century [22]. A limitation of the study is that 22 patients were operated with a lateral transgluteal approach which was than changed to a direct anterior minimal invasive approach during the study period [23], which showed no statistical difference. There might be some patient bias in this study, since the elder and rather frail patients received the cemented twinSys stem including also femoral neck fractures. The healthier and younger patients (*n* = 185) during the observed time period were operated with the uncemented twinSys stem. Furthermore, as we are a teaching hospital, a substantial number of operations have been performed by residents what might, in parts, explain our higher rate of infection and death during the course of follow-up [24–27].

### Radiological outcome

As expected radiological changes on plain radiographs were scarce concerning osteolysis and debonding, which is the expected course of well cemented stems with short-term follow-up. Cementing quality has been shown to be crucial for long-term survival of cemented implants [28]. The overall cementing quality in our series was good especially in the setting of a teaching hospital with residents performing 36 % percent of the surgery in this series. Anyhow we did not reach the cemeting quality of highly specialised centers [29].

### EBRA-FCA analysis

A limitation of our radiological analysis is that 27 stems had no complete radiological follow-up. With a mean age of 79 years at the time of surgery the study group was rather old and patients unwilling to come in case of a symptom free follow-up. 57 out of 73 stems with a complete follow-up the number of radiographs suitable for an EBRA-FCA analysis is a bit higher than reported in the literature [3].

In the literature differerent cut-off values for early subsidence being indicative for later aseptic loosening are described. Freeman et al. reported a later failure of the femoral component if early migration was above 1.2 mm/year in the first 2 years with a sensitivity of 78 % measured on plain radiographs [1]. Similar values on plain radiographs were supported by Walker et al. stating a long-term survival rate of less than 5 % for Charnley and Stanmore stems, that showed a subsidence of <2.6 mm in the first 2 years [4]. Measurements for cemented shape-closed stems (Lubinius and Spectron SP1) on normal radiographs has a lower accuracy compared to software based methods [30]. Kärrholm et al. [2] defined 1.2 mm in the first 2 years as the cut-off value for later aseptic loosening of the stem (Lubinius SP1, shape-closed) using the more accurate RSA method (accuracy 0.2 mm) while De Vries et al. analysed 15 different stem designes and reported 1.24 mm for straight polished cemented stems as the cut-off for later aseptic loosening [31]. RSA is the method with the highest precision but as a short-coming needs special radiographs, implants (markers) and is therefore not suitable for larger series [32]. The EBRA-FCA method used in this study has the advantage that routine clinical radiographs can be used to analyse large series [33] but lacks the accuracy of RSA with a detection limit for subsidence of 0,2 mm. Krismer et al. [3] reported a subsidence >1.5 mm in the first 2 years as the cut-off value for later aseptic loosening for cemented Müller straight stems. The Müller straight stem is a shape closed stem [34], which by design should not subside. In contrast to shape-closed stems with a force-closed philosophy as the twinSys are intended to show some subsidence [13, 35]. We measured a mean subsidence of 0.33 mm which is clearly below all publishedcut-off values independent from the respective method.

Anyhow 9 stems in our series showed an early subsidence between 1–1.5 mm which is of concern. These stems have to be closely monitored, whether this is the final position or if further migration will occur. We found only two stems with a subsidence > 1.5 mm indicative for later aseptic loosening. One of these two just subsided 1.5 mm lacking any other radiological abnormalities. The other one subsided 3.1 mm after an early infection 4 weeks after surgery and was treated with DAIR. It is rather unlikely that subsidence was caused by a persisting chronical infection as the patient was free of symptoms of infection during further follow-up, but intraoperative manipulation of the stem during DAIR might be a potential explanation for the excessive subsidence.

## Conclusions

The cemented twinSys stem showed excellent clinical and radiologic short term results at 2 years follow-up. We found a mean subsidence of 0.44 mm which is clearly below the 1.5 mm published in the literature being the cut-off valuefor later aseptic loosening of the stem, the cemented twinSys stem seems to be a reliable implant even in the setting of a teaching hospital resulting in inferior cement mantle quality than in specialised centers. Further follow-up of this patient group as well as larger series including also younger and more active patients can prove the longevity of the stem.

## Abbreviations

ARR: Acetabular reinforcement ring
DAIR: Debridement and implant retention
EBRA-FCA: Einzelbildröntgen analyse- femoral component analysis
EKNZ: Ethik Kommission Nordwestschweiz = responsible local ethik commission in northwest Switzerland
HHS: Harris hip score
KM: Kaplan-Meier
PE: Polyethylen
RSA: Roentgen stereophotogrammetric analysis
THA: Total hip arthroplasty
VAS: Visual analog scale
